# Phenolic Acids and Amaryllidaceae Alkaloids Profiles in *Leucojum aestivum* L. In Vitro Plants Grown under Different Light Conditions

**DOI:** 10.3390/molecules28041525

**Published:** 2023-02-04

**Authors:** Emilia Morańska, Magdalena Simlat, Marzena Warchoł, Edyta Skrzypek, Piotr Waligórski, Dominique Laurain-Mattar, Rosella Spina, Agata Ptak

**Affiliations:** 1 Department of Plant Breeding, Physiology and Seed Science, University of Agriculture in Krakow, 31-140 Krakow, Poland; 2 The Franciszek Górski Institute of Plant Physiology Polish Academy of Sciences, 30-239 Krakow, Poland; 3 Institut National de Recherche pour l’Agriculture, l’Alimentation et l’Environnement, Laboratoire Agronomie Environnement, Université de Lorraine, 54000 Nancy, France

**Keywords:** light-emitting diodes, *Leucojum aestivum* L. in vitro culture, photosynthetic pigments, sugar, phenolic compounds, galanthamine, lycorine

## Abstract

Light-emitting diodes (LEDs) have emerged as efficient light sources for promoting in vitro plant growth and primary and secondary metabolite biosynthesis. This study investigated the effects of blue, red, and white-red LED lights on plant biomass growth, photosynthetic pigments, soluble sugars, phenolic compounds, the production of Amaryllidaceae alkaloids, and the activities of antioxidant enzymes in *Leucojum aestivum* L. cultures. A white fluorescent light was used as a control. The plants that were grown under white-red and red light showed the highest fresh biomass increments. The blue light stimulated chlorophyll *a*, carotenoid, and flavonoid production. The white-red and blue lights were favourable for phenolic acid biosynthesis. Chlorogenic, *p*-hydroxybenzoic, caffeic, syringic, *p*-coumaric, ferulic, sinapic, and benzoic acids were identified in plant materials, with ferulic acid dominating. The blue light had a significant beneficial effect both on galanthamine (4.67 µg/g of dry weight (DW)) and lycorine (115 µg/g DW) biosynthesis. Red light treatment increased catalase and superoxide dismutase activities, and high catalase activity was also observed in plants treated with white-red and blue light. This is the first report to provide evidence of the effects of LED light on the biosynthesis of phenolic acid and Amaryllidaceae alkaloids in *L. aestivum* cultures, which is of pharmacological importance and can propose new strategies for their production.

## 1. Introduction

*Leucojum aestivum* L. belonging to the Amaryllidaceae family is an important medicinal plant mainly due to its alkaloid content, such as galanthamine and lycorine [1]. Galanthamine has been used in the treatment of Alzheimer’s disease since 2001 [2], while lycorine is at the clinical trial stage for its anticancer and antiviral properties [3]. Studies have shown that lycorine also has the potential to combat SARS-CoV-2 infection due to its antiviral activity [4]. Considering the increasing number of people suffering from Alzheimer’s disease, the currently existing sources of galanthamine are not profitable enough; thus, there is a search for biotechnological methods as an alternative to the existing methods of obtaining this alkaloid. It has been demonstrated that in vitro *L. aestivum* cultures can be a valuable source of galanthamine and other Amaryllidaceae alkaloids. Detailed information on this subject can be found in Laurain-Mattar and Ptak [1], Georgiev et al. [5], and Koirala et al. [6]. It should be noted, however, that the galanthamine content obtained from in vitro cultures is not yet satisfactory as a method of obtaining alkaloids to be implemented in production. Hence, various factors, mainly abiotic elicitors, which can enhance in vitro biosynthesis of Amaryllidaceae alkaloids are being tested [1,7,8]. Light is one of the important abiotic elicitors influencing plant growth and the production of metabolites for in vitro cultures [9]. Tubular fluorescent lamps are commonly used for in vitro cultures. Light-emitting diodes are a potential alternative and new light source for in vitro culturing due to their spectral composition control, narrow bandwidth, low amounts of thermal emissions, low degradation, and long life [10].

Regarding in vitro cultures of *L*. *aestivum*, only the effects of white fluorescent light and darkness on galanthamine biosynthesis have been investigated [11]. In vitro studies on another species of this family, *Lycoris longituba*, have shown that blue LED light stimulates the biosynthesis of galanthamine, lycorine, and lycoramine in seedling cultures [12]. However, the responses of the plants vary considerably to different light treatments, with no specific pattern among the various species. For example, red light stimulates flavonoid biosynthesis in vitro in shoots of *Myrtus communis* [13] and callus cultures of *Sylibum marianum* [14], as well as isosteroidal alkaloids in callus cultures of *Fritillaria cirrhosa* [15], whereas blue light increases hypericin content in root cultures of *Hypericum perforatum* [16]. The combination of red and blue lights (1:1) increases biomass accumulation and protein content in callus cultures of *Hyoscyamus reticulatus*, as well as increasing biomass accumulation, anthocyanin and 20-hydroxyecdysone content in plants of *Pfaffia glomerata* in vitro [17,18]. Interestingly, the combination of green, red, and blue lights promotes biomass growth and the production of glucosinolates in *Nasturtium officinale* shoot cultures [19], while white light stimulates chicoric acid accumulation in callus cultures of *Ocimum basilicum* [20].

Generally light, as an elicitor, can control primary metabolism through carbohydrate production, which is directly related to the photosynthetic process [21], and secondary metabolism: phenolic compounds, essential oils, alkaloids, and other compounds [19,22,23,24]. It is known that stress induced by elicitors results in the activation of several defence-related genes or the inactivation of non-defence-related genes, and the expression of enzymes whose information can be used to ascertain the biosynthetic pathways of metabolites [25]. The biosynthesis of stress-inducible alkaloids is a natural adaptive response to abiotic and biotic stress factors [9]. In vitro stress caused by the action of elicitors is also often accompanied by increased biosynthesis of soluble sugars and phenolic compounds [26,27,28]. Soluble carbohydrates are synthesised, for example, in response to osmotic stress, protecting cellular members and improving survival under stress conditions [29]. Biotic elicitors, such as pathogen infection, also produce changes in the distribution and metabolism of carbohydrates in plants [30]. Red plus blue LEDs [31] stimulate carbohydrate biosynthesis in vitro in *Doritaenopsis* plants, as does white LED light in *N*. *officinale* shoots [19]. In Hassanpour’s [18] study, different LED lights enhanced total sugar content in the *H*. *reticulatus* callus cultures exposed to different LED spectra compared to dark conditions. A blue LED light promoted phenolic compound synthesis in callus cultures of *Vitis davidii* [32], *Rehmannia glutinosa* plants [33], and *H*. *perforatum* root [16] cultures; red light in shoots of *M*. *communis* [13]; and red-blue and red-green-blue lights in callus cultures of *Camellia japonica* [34]. Phenolic compounds are specialised metabolites produced by plants mainly for their growth, development, and protection during abiotic and biotic stresses [35]. Phenolic compounds contribute significantly to the plant’s resistance to pests, pathogens, and environmental stresses. Under stressful conditions, phenols accumulate drastically in the plant for it to survive. Phenolic compounds act as antioxidants and combat these stress states in plant cells. These compounds can detoxify reactive oxygen species in plants and have the capacity to cure many human diseases caused by oxidative damage and aging. Hence, there is currently significant interest centred on their potential uses in medicine [36] and agriculture [37]. 

The aim of the study was to investigate whether LED light quality causes a stress response in vitro in *L*. *aestivum* plants, which can be manifested by increased biosynthesis of sugars and phenolic compounds (phenolic acids and flavonoids), as well as the activities of antioxidant enzymes (catalase (CAT), peroxidase (POD) and superoxide dismutase (SOD)); thus, whether it affects the biosynthesis of Amaryllidaceae alkaloids. The presented research may provide new and valuable information for a further strategy concerning the possibility of obtaining valuable specialised metabolites, such as phenolic compounds and Amaryllidaceae alkaloids from in vitro cultures of *L. aestivum*.

## 2. Results and Discussion

### 2.1. Effect of Light Quality on In Vitro Plant Growth

**Figure 1:**
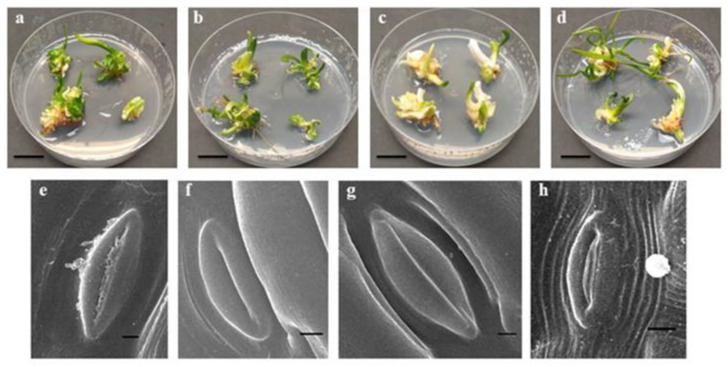
Growth of *L. aestivum* plants (bar = 2 cm), and stomata leaf (bar = 100 µm) appearance after four weeks of culturing under different light conditions: (**a**,**e**) white (F) (control), (**b**,**f**) blue (L), (**c**,**g**) red (L), (**d**,**h**) white (L)-red (L). F: fluorescence, L: LED light.

The *L*. *aestivum* somatic embryos converted into plants after four weeks of growth, regardless of the applied light conditions. However, it was observed that plants obtained under mixed LED lights (white and red, 1:1) were the most abundant and developed narrow and long leaves with light green colouring. The plants obtained under blue LED light were the least abundant compared to plants obtained under the other tested conditions. Moreover, they produced slightly thickened leaves with either a light yellow colour and a green spot on the leaf tip or were completely unstained (Figure 1). LED lights can affect morphogenesis, in vitro plant growth, and can have a significant effect on the biomass of the plant [38].

**Figure 2:**
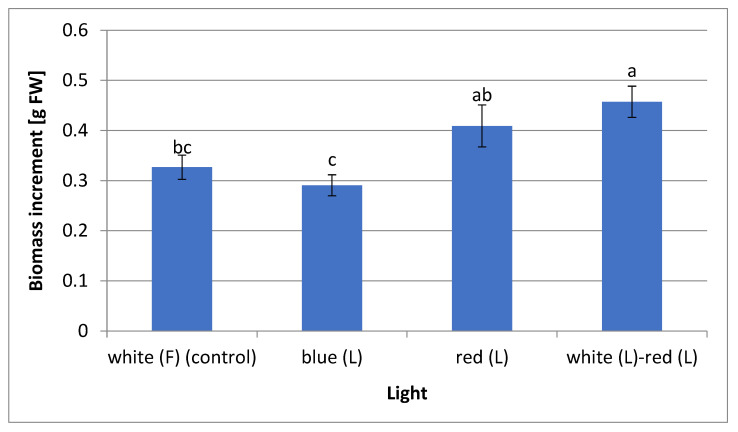
Biomass increments of *L. aestivum* plant cultures depending on the applied lighting conditions. Values are expressed as mean ± SD (*n* = 10). Different letters indicate a significant difference at *p* < 0.05 according to ANOVA and Duncan’s tests. F: fluorescence, L: LED light, FW: fresh weight.

The mixed white and red (1:1) LED light stimulated the greatest increase in the fresh biomass of the *L. aestivum* plants (0.46 g fresh weight: FW). Slightly lower values were recorded for the plant material obtained with a red LED light (0.41 g FW). The plants that grew under the blue LED and white fluorescent lights (control) showed the lowest biomass increases (0.29 g and 0.33 g FW, respectively) (Figure 2). In the Cioć et al. [13] study, blue light, contrary to the presented research, had a positive effect on obtaining *Myrtus communis* shoots, characterised by the highest fresh biomass. Several studies have shown that the combination of different wavelengths of light LEDs can promote plant growth. Most often, various proportions of red and blue lights are used. Silva et al. [17] found that a mixture of red and blue lights (1:1) increased biomass accumulation in plants of *Pfaffia glomerata* in vitro, while Hung et al. [39] showed that a combination of 50% red and 50% blue LED lights increased shoot and root biomass of *Vaccinium corymbosum* plants in vitro. Using mixed light, Hassanpour [18] discovered that a mixture of red and blue lights (75%:25%) was optimal for obtaining the maximum biomass of *Hyoscyamus reticulatus* callus cultures. Li et al. [12] also indicated that red and blue lights (1:2) increased the biomass of *Lycoris longituba* seedlings. A combination of white and red LED lights is less frequently utilised. As in the current study, Simlat et al. [40] showed a positive effect of this mixed light on the fresh biomass of *Stevia rebaudiana* plants obtained from in vitro seedlings.

### 2.2. Effects of Light Quality on Photosynthetic Pigment Content and Stomata Appearance

In the present study, blue LED light stimulated chlorophyll *a* biosynthesis in *L*. *aestivum* plant cultures (16.00 µg/g FW). Slightly lower amounts of chlorophyll *a* have been reported in white fluorescent light-derived plants (14.13 µg/g FW), while the lowest accumulation of this pigment was noted using mixed (white and red, 1:1) light and red light (8.51 µg/g FW and 13.06 µg/g FW, respectively) (Table 1). However, in the present study, no significant difference was found in the content of chlorophyll *b* in the plant material from different light conditions (Table 1). The accumulation of this pigment was maintained at an average level of 11.68 µg/g FW. The content of carotenoids in the plants growing in the tested lighting variants ranged from 0.37 µg/g FW for the mixed white and red to 1.82 µg/g FW for the blue LED light and 2.24 µg/g FW for the white fluorescence light (Table 1).

Red and blue lights are the most effectively utilised wavelengths for plant photosynthesis because the absorption spectrum of the photosynthetic pigments mainly focuses on the red (600–700 nm) and blue (400–500 nm) light spectrums. Red light can regulate the operation of the plant’s photosynthetic system and the transport of assimilates, while blue light is involved in stomatal opening [41]. Blue and red LED lights stimulate the biosynthesis of chlorophyll *a* in the shoot cultures of *Nasturtium officinale* [19]. Blue LED light promotes chlorophyll biosynthesis in *Vaccinium corymbosum* [39]. In turn, during the in vitro growth of *Lycoris longituba* seedlings, Li et al. [12] observed that the use of red and blue LED lights (1:2) had a positive effect on the total chlorophyll content. Klimek-Szczykutowicz et al. [19] showed that white LED had a positive effect on chlorophyll *a* content in *N*. *officinale* cultures, while white fluorescent light, contrary to the results of this study, inhibited the biosynthesis of chlorophyll *a* and *b*. Moreover, white fluorescent light promoted chlorophyll *a* and *b* accumulation in *Stevia rebaudiana* plants in vitro, whereas red light, as in the present research, had a negative effect on the content of the pigments [40]. Conversely, Zheng and van Labeke [42] did not notice any significant effects of the light quality on the photosynthetic pigments in the leaves of *Ficus benjamina*. 

Several authors have highlighted that the effect of light is species dependent, and that the biosynthesis of photosynthetic pigments may also be influenced by the intensity of the lighting. Similarly, being too highly irradiated can destroy the photosynthetic apparatus and lower pigment synthesis [43], while irradiation that is too low does not promote efficient photosynthesis [44]. Zhang et al. [45] indicated that 100 μmol m^−2^ s^−1^ blue LED light was effective in increasing carotenoid content in the juice sacs of *Citrus unshiu* obtained in vitro. In contrast, 50 μmol m^−2^ s^−1^ blue LED light treatment was effective in inducing carotenoid accumulation in *Citrus sinensis*. In the present study, one light intensity (60 µmol m^−2^ s^−1^) was used. 

The action of light with different spectral ranges modifies the reactions of stomatal cells [46]. In this study, leaves from the *L. aestivum* plants grown under both white fluorescent and blue LED lights were open and possessed properly developed stomata (Figure 1e, f). However, the stomata of the plants exposed to the red LED light remained closed (Figure 1g). Blue light is important in chlorophyll biosynthesis, stomatal opening, enzyme synthesis, chloroplast maturation, and photosynthesis [47]. The stomata also remained open in the leaves of the plants grown under mixed LED lights (white and red); however, some were incorrectly developed (Figure 1h). Simlat et al. [40] made analogous observations in *S*. *rebaudiana* plants, where blue LED light, similar to white fluorescent light, promoted the opening of the stomata compared to the red LED light alone.

### 2.3. Effects of Light Quality on Sugar, Phenolic Compounds Content, and Antioxidant Enzyme Activities

Light is used by plants as an energy source; however, it can also promote stress in plants and modulate stress responses [48]. The current research revealed several biochemical, physiological, and genetic markers of the stress response. Among them, sugars and phenolic compounds play a key role in plant defence responses to various stress factors [49]. In this presented research, the light quality did not affect the total sugar content in *L. aestivum* plants cultured in vitro (Table 2). The sugar content in all plant materials remained at an average level of about 71.8 mg/g DW. Similarly, Simlat et al. [40] found no correlation between the level of sugar accumulation and the LED light quality used for the growth of *S. rebaudiana* seedlings. Conversely, carbohydrate biosynthesis in *Doritaenopsis* plants, in vitro, has been stimulated by red plus blue LEDs [31] and by white LED light in *N. officinale* shoots [19].

LED light had no significant effect on the total amount of phenolic compounds in *L. aestivum* plants, which was on average 50.03 mg/g DW (Table 2). On the other hand, white and blue LED lights stimulated the accumulation of phenolic compounds in *Lachenalia* sp. adventitious shoots [50]. Hashim et al. [41] presented review studies on the effects of LED light in stimulating the biosynthesis of phenolic compounds in different species. However, little research has been conducted on the biosynthesis of phenolic compounds in the in vitro cultures of Amaryllidaceae plants. The total content of phenolic compounds has been determined only in *L. aestivum* in vitro plants grown in the presence of various sugars within the medium [26]. 

In the present study, we showed that light affected the total flavonoid content in *L. aestivum* cultures (Table 2). The highest flavonoid content was observed in plants treated with white fluorescent light (0.40 µg/mg DW), while slightly lower amounts were observed in plants treated with the mixed white and red LED lights (0.36 µg/mg DW) and blue light (0.32 µg/mg DW), respectively. Flavonoids, which have a polyphenolic structure are important secondary metabolites in plants. They have different biological functions during different stages of plant growth and development. Flavonoids are also known to have a protective role against stress conditions, such as pathogen infections, UV-B radiation, drought, cold, and salinity [51,52]. To the best of our knowledge, no studies are available on the effects of LED light on the flavonoid content of in vitro cultures of *L. aestivum*, although studies by Manivannan et al. [53] have shown that red LED light stimulated flavonoid biosynthesis in *Rehmannia glutinosa* in vitro cultures.

**Figure 3:**
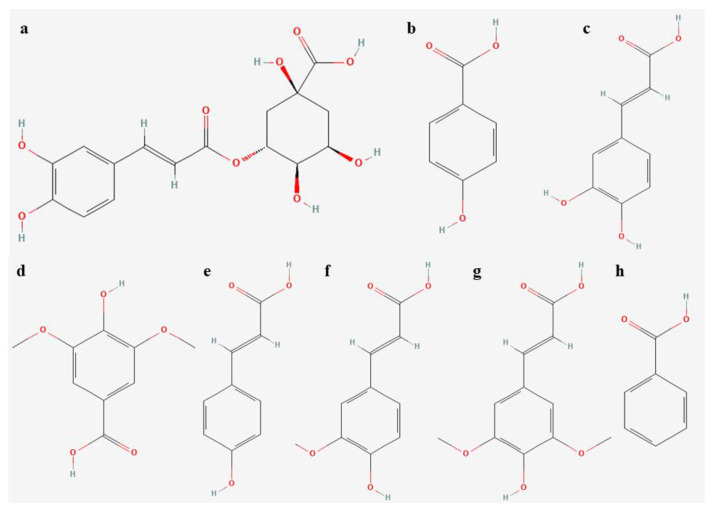
Chemical structure of phenolic acids isolated in extracts of *L. aestivum* in vitro plants (**a**) chlorogenic acid, (**b**) *p*-hydroxybenzoic acid, (**c**) caffeic acid, (**d**) syringic acid, (**e**) *p*-coumaric acid, (**f**) ferulic acid, (**g**) sinapic acid, (**h**) benzoic acid.

In terms of other polyphenolic compounds, we found eight phenolic acids in *L. aestivum*, including chlorogenic, *p*-hydroxybenzoic, caffeic, syringic, *p*-coumaric, ferulic, sinapic, and benzoic acids (Figure 3 and Table 3).

To date, the in vitro content of phenolic acids in the cultures of any plants of the Amaryllidaceae family has not been determined. However, Nikolova and Gevrenova [53] demonstrated the presence of phenolic acids in leaves from five species of the Amaryllidaceae family grown in natural conditions. *L. aestivum* plants grown in vitro synthesised the largest amount of ferulic acid (average: 3.07 ng/mg DW, regardless of the light conditions used) (Table 3).

Ferulic acid was also dominant in *L. aestivum* plants grown in natural conditions [53]. Ferulic acid possesses diverse biological functions–for example, it has strong antioxidant activity and is a promising molecule for the treatment of vascular disorders [54]. Notably, apart from ferulic acid, only small amounts of *p*-coumaric and vanillic acid were detected in the plants grown in natural conditions, which may suggest that *L. aestivum* plants grown in vitro could provide a richer source of phenolic acids than those grown in natural conditions [53].

In our in vitro experiments, the light quality had a significant impact on the production of phenolic acids in *L. aestivum* plants (Table 3). The mixed white-red LED lights stimulated the biosynthesis of *p*-hydroxybenzoic, caffeic, syringic, *p*-coumaric, and ferulic acids, while blue LED light had a positive effect on *p*-hydroxybenzoic, sinapic, and benzoic acid production. The highest total content of all detected phenolic acids was extracted from plants grown in the mixed white and red LED lights (13.65 ng/mg DW) and blue light (13.05 ng/mg DW) conditions, whereas the lowest was found in plants grown under the white fluorescence light (10.38 ng/mg DW). Contrary to our study, for in vitro cultures of *Myrtus communis*, the lowest amount of phenolic acid was observed in plants grown under blue LED light [13]. The white LED light stimulated chicoric acid biosynthesis in the in vitro cultures of the *Ocimum basilicum* [20].

The red LED treatment increased CAT (1.8-fold) and SOD (2.0-fold) activities in the *L. aestivum* plants compared to the cultures grown under the fluorescent lamps (Table 4). We also observed high, but slightly lower, CAT activity using mixed (white and red) and blue LED light (1.3 fold), but the LED light had no impact on POD activity (Table 4).

This indicates that light-stressed *L. aestivum* plants exhibit specific responses to antioxidant enzyme activities. Research has shown that red light generates an antioxidant response in lettuce plants grown in a greenhouse [55]. Conversely, blue LED light increases CAT activity in the shoots of *Dracocephalum forrestii* in vitro [56], alongside the seedlings of *S. rebaudiana* [40] and *L. longituba* [12]. Studies have revealed that when plants are exposed to stressful conditions, their antioxidant systems play key protective roles by increasing the activities of the antioxidant enzymes such as POD, CAT, and SOD. This is an important strategy for reducing reactive oxygen species formed during stress reactions. SOD is usually considered the first line of defence against oxidative stress [57]. SOD catalyses the dismutation of superoxide to O_2_ and H_2_O_2_, CAT removes the resulting H_2_O_2,_ while POD is the key enzyme responsible for H_2_O_2_ scavenging during oxidative stress in plants [58,59]. Interestingly, in the present study, red LED light increased the activities of CAT and SOD and promoted the biomass growth of *L. aestivum* plants (Figure 2) — which may suggest that red light causes some stress to in vitro cultures of *L. aestivum*, yet, at the same time, the cell detoxification system is quite effective. It is also worth noting that high CAT activity was recorded using the mixed (white and red) and blue LED lights. Under the same conditions, high amounts of flavonoids and the highest contents of phenolic acids were also observed (Table 2, Table 3). Therefore, it can be assumed that the stress, which occurred under the influence of these lights caused changes in the secondary metabolism pathways that were manifested by the increased synthesis of flavonoids and phenolic acids.

### 2.4. Effect of Light Quality on Amaryllidaceae Alkaloid Biosynthesis 

**Figure 4:**
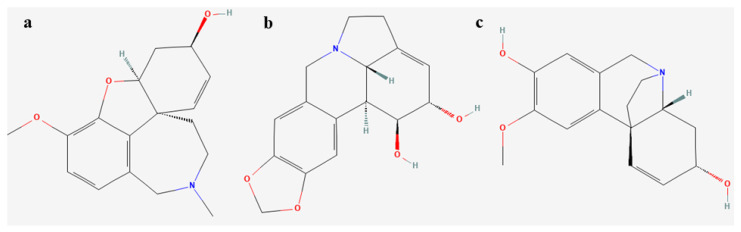
Chemical structure of Amaryllidaceae alkaloids isolated in *L. aestivum* in vitro plants (**a**) galanthamine, (**b**) lycorine, (**c**) demethylmaritidine.

The qualitative analysis of alkaloids from *L. aestivum* plants obtained under different light conditions was performed using gas chromatography–mass spectrometry (GC-MS). In plants obtained from somatic embryos under the influence of the blue LED and white fluorescent lights, the presence of demethylmaritidine and lycorine was noted, whereas, in the plants derived from the red LED light, none of the alkaloids were detected (Figure 4, Table 5).

Studies have shown that light influences the synthesis of secondary metabolites in plants, with blue light most often stimulating biosynthesis and red light having a negative effect [60]. It is also worth noting that up to two alkaloids were identified in *L. aestivum* plants growing under different light conditions (Table 5).

This may be because the research used very young plants, obtained four weeks after placing the somatic embryos on the medium for their conversion. In previous studies, 0–4 alkaloids were detected in callus tissues and 6 alkaloids in the 12-month-old *L. aestivum* plants [26,61]. This confirms the observations that the biosynthesis of secondary metabolites may relate to the level of tissue organization [62,63].

**Figure 5:**
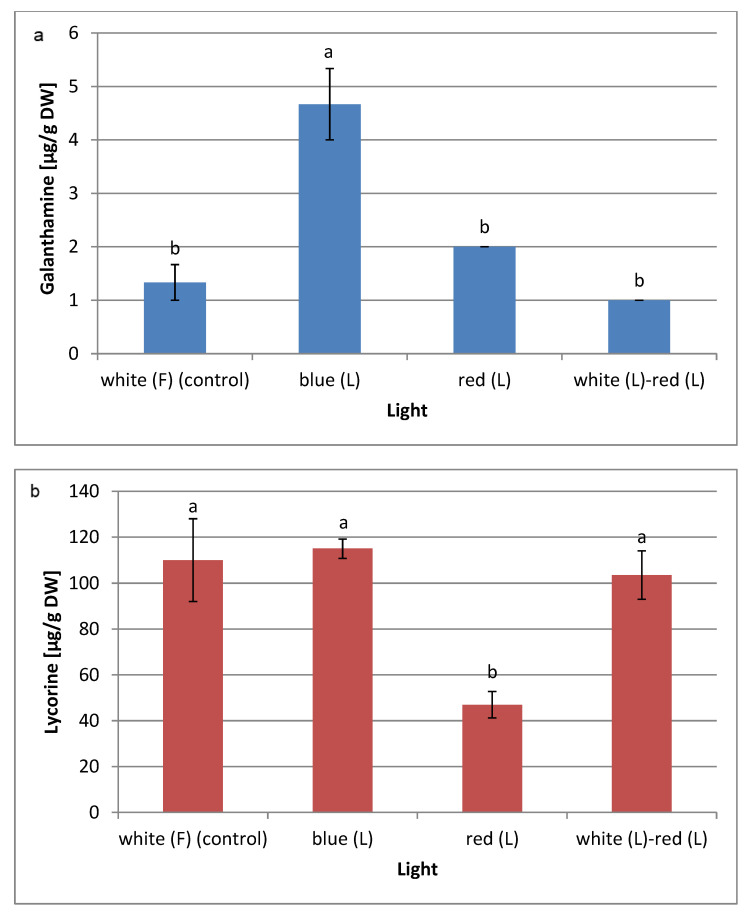
Effect of different light conditions on (**a**) galanthamine and (**b**) lycorine contents determined by LC-MS in *L. aestivum* in vitro plants. The results are means of 3 replicates (*n* = 3). Error bars represent ± SD. Different letters indicate a significance difference at *p* < 0.05 according to ANOVA and Duncan’s test. F: fluorescence, L: LED light, DW: dry weight.

It is worth noting that the liquid chromatography–mass spectrometry (LC-MS) used for the quantification of the alkaloids from the *L. aestivum* plants allowed for the detection of galanthamine and lycorine in all the samples studied, including those in which they were not detected by the GC-MS analysis (Figure 5).

The highest galanthamine content (4.67 µg/g DW) was observed in plants obtained under the blue LED light condition. This content was 3.5 times higher than in the control plants grown under the white fluorescent light. The highest lycorine content (115 µg/g DW) was also recorded in plants grown under the blue LED light, although this content did not differ statistically between the amounts of lycorine obtained in plants derived from the white fluorescent light and the mixed white and red LED lights. On the other hand, a negative effect of the red light on the biosynthesis of lycorine was noted. Regarding studies on the in vitro cultures of *L. aestivum*, shoots cultured under white fluorescent light have produced twice as much galanthamine as those cultured in darkness [11]. The effect of the LED light on the biosynthesis of Amaryllidaceae alkaloids in *L. aestivum* cultures has not been tested thus far. On the other hand, in vitro studies of *L. longituba* seedlings showed that blue LED light, similar to the present study, stimulated the production of galanthamine and lycorine, while red LED light had a negative effect. Blue light also induces the expression of Amaryllidaceae alkaloid biosynthesis pathway genes in these cultures [12]. In the Park et al. [64] study, blue LED light promoted ginsenoside biosynthesis in *Panax ginseng* root cultures. However, contrary to our results, the red LED light favoured the production of alkaloids in *Fritillaria cirrhosa* callus cultures [15].

Our study clearly showed that the blue LED light promoted the biosynthesis of Amaryllidaceae alkaloids, flavonoids, and phenolic acids. The *L. aestivum* plants grown under the blue LED light also showed an increase in CAT activity and inhibited the plant’s biomass (Table 2, Table 3, Figure 2, and Figure 5a). These results imply that the light stress alleviation mechanism rendered the elicitation of secondary metabolites in the in vitro cultures of *L. aestivum*. Elicitation is one of the most promising methods to increase secondary metabolite biosynthesis through stress induction. It involves the manipulation of metabolite and biochemical pathways. However, the presence of stress means that the plant grows in a non-optimal or poor state that negatively affects it [65]. The effect of light on secondary metabolite biosynthesis and in vitro plant growth may, therefore, be genotype dependent. Ardelan et al. [66] showed that blue LED light stimulated the biosynthesis of phenolic compounds and flavonoids, while it did not affect the growth of basil plants. In contrast, according to Cioć et al. [13], blue LED light had a positive effect on *Myrtus communis* shoot biomass and a negative effect on myricetin biosynthesis. Blue light can increase chlorophyll content, which results in increased photosynthesis and, thus, more plant biomass. However, in the case of bulbous plants, the positive effect of red or mixed blue-red light on the growth and formation of bulbs is often observed [12]. This confirms our observations, whereby the white-red and red lights were better for *L*. *aestivum* plant biomass growth than the blue light. In the future, a two-step cultivation strategy should be considered for the mass production of Amaryllidaceae alkaloids in *L. aestivum* in vitro cultures. The first step should be biomass accumulation, while the second should be the high production of alkaloids.

Several authors have also indicated that the use of mixed LED lights (blue and red), in the right proportions, may affect the increase of biomasses and the production of secondary metabolites. Costa et al. [67] used blue and red lights in a 3:1 proportion, respectively, to increase the biomass and bergapten production of *Brosimum gaudichaudii* seedlings. Conversely, the combination of blue and red lights, in a ratio of 2:1, was optimal for *L*. *longituba* seedling growth, while the use of the blue light alone favoured the biosynthesis of Amaryllidaceae alkaloids [12]. 

## 3. Materials and Methods

### 3.1. In Vitro Experimental Cultures

*Leucojum aestivum* L. somatic embryos in the torpedo stage were used as plant material in this research. To obtain somatic embryos, leaf fragments were isolated from bulbs, chilled for 12 weeks at 5 °C, and placed in solid Murashige and Skoog (MS) [68] medium containing 25 µM picloram (4-amino-3, 5, 6-trichloropicolinic acid, Sigma-Aldrich, St. Louis, MO, USA) and 0.5 µM BA (6-benzyladenine, Sigma-Aldrich, St. Louis, MO, USA). After 12 weeks of culturing, the embryogenic callus was separated from primary explants and multiplied during eight weeks in the medium with the addition of 5 µM picloram and 0.5 µM BA. Somatic embryos were induced in the same medium. The detailed procedure was described earlier by Ptak et al. [7].

The somatic embryos were grown in a solid MS [68] medium enriched with 5 µM zeatin. The pH was adjusted to 5.8. The plant material was treated with four different light quality combinations: white fluorescent lamp (390–760 nm, OSRAM Fluora 36W/77, Munich, Germany) as a control, blue LED (445 nm), red LED (638 nm), and mixed: white (420 nm) and red LED (1:1) (Snijders Scientific, Netherlands). Cultures were maintained for four weeks in a climatic chamber (MCA 1600, Snijders Scientific, Netherlands) at 25 ± 1 °C (day/night) and 70% relative humidity, different light sources (16/8 h photoperiod (day/night)) were used and PPFD was maintained constant at 60 µmol m^−2^ s^−1^ for all treatments. About 4 g of somatic embryos were placed in each petri dish. The experiment was set up in 10 replicates. In total, about 200 embryos were used for each of the combinations. After four weeks of culturing, the increase in the FW of plant material was determined.

### 3.2. Scanning Electron Microscopy

To observe the appearance of the stomata in the leaf blades of the regenerated plants, the leaves were fixed with 2.5% (*w*/*v*) glutaraldehyde (Merck SA, Darmstadt, Germany) in 0.1 M phosphatic buffer (Merck SA, Darmstadt, Germany) at pH 7.2–7.4 for 15 min. They were dehydrated using a graded series of ethanol (Sigma-Aldrich, St. Louis, MO, USA) (15–100% *v*/*v*) and acetate (100%) (Sigma-Aldrich, St. Louis, MO, USA). Samples were critical point dried with liquid CO_2_ (Air Liquide, Krakow, Poland) in a critical point dryer (Type E3100 Industrial LADD, Kettering, Ohio, USA) and then coated with gold using a sputter coater (Jeol JFC-1100E, Akishima, Japan). Finally, samples were scanned using a scanning electron microscope (Jeol model JSM 5410, Akishima, Japan), according to Ptak et al. [69].

### 3.3. Determination of Photosynthetic Pigments

For each analysis, 100 mg of fresh plant tissue was extracted in 1.5 mL of 80% aqueous ethanol. The homogenate was stored overnight at 4 °C, in the dark, and then centrifuged at 2800 rpm for 5 min (Eppendorf Centrifuge 5702 R, Hamburg, Germany). Next, 20 µL supernatant was mixed with 0.2 mL 80% aqueous ethanol (Avantor Performance Materials Poland SA, Gliwice, Poland). The photosynthetic pigments contents (chlorophyll *a*, chlorophyll *b*, and total carotenoids) were determined by measuring absorbances at 470, 648, and 664 nm using a spectrophotometer (Synergy II, Bio-Tek, Winooski, VT, USA) and calculating according to Lichtenthaler & Wellburn [70].

### 3.4. Determination of Soluble Sugars

Lyophilised plant tissue (5 mg) was extracted with 1.5 mL of 80% aqueous ethanol and then centrifuged at 2800 rpm for 10 min. The amounts of total soluble sugars were estimated by the phenol–sulphuric procedure explained by Dubois et al. [71]. The reaction mixture contains 10 µL of supernatant, 0.2 mL of distilled H_2_O, 0.2 mL of 5% phenol (Avantor Performance Materials Poland SA, Gliwice, Poland), and 1 mL of 96% H_2_SO_4_ (Avantor Performance Materials Poland SA, Gliwice, Poland). After incubating for 30 min, the absorbance was measured spectrophotometrically at 490 nm. The amounts of soluble sugars were determined against a glucose standard curve and expressed in milligrams per gram of DW.

### 3.5. Determination of Total Phenolic Compounds

To assay the total phenolic compounds according to the Folin–Ciocalteu method [72], 5 mg of lyophilised plant tissue was ground with 80% aqueous ethanol in 1.5 mL tubes. After 20 min of centrifugation at 2800 rpm, the 20 µL of supernatant was mixed with 1 mL of H_2_O, 0.5 mL of 20% Na_2_CO_3_ (Merck SA, Darmstadt, Germany) and the 125 µL of Folin–Ciocalteu reagent (Chempur, Piekary Śląskie, Poland) (diluted 1:1 with distilled water before use). After 20 min of incubation, the absorbance (*λ* = 760 nm) of the samples was estimated spectrophotometrically. The total phenolic content was calculated as milligrams of chlorogenic acid per gram of DW.

### 3.6. Determination of Flavonoids and Phenolic Acids

#### 3.6.1. Extraction

Lyophilised and powdered samples (about 5 mg) were extracted twice with 100 µL ethanol/water (1:1) and 0.01% HCl (Chempur, Piekary Śląskie, Poland). During extraction, samples were sonicated (10 min) and centrifuged for 10 min at 15,000 rpm. Supernatants were combined.

#### 3.6.2. Total Flavonoids Assay

The total flavonoid content was measured by a spectrophotometer according to Ramos et al. [73]. Ten microliters of the extract was mixed with 150 µL of 2% AlCl_3_ (Sigma-Aldrich, St. Louis, MO, USA) in water. After 10 min of incubation, the absorbance was measured at 420 nm. A calibration curve of quercetin standards (Sigma-Aldrich, St. Louis, MO, USA) was prepared (between 0.5 mg/mL and 0.005 mg/mL). 

#### 3.6.3. Analyses of Phenolic Acids 

Analyses were carried out using the HPLC system (Agilent Technologies, Santa Clara, California, USA), consisting of an ACQUITY BEHC18 1.7 μm, 2.1 × 100 mm analytical column, the solvents used were water with 0.1% formic acid (A) and acetonitrile with 0.1% formic acid (B) (Merck SA, Darmstadt, Germany). A gradient was set from 15% to 90% B in 8 min. The HPLC system used was Agilent Technologies 1260 with a binary pump and QQQ 6410 mass spectrometer as a detector; 2 µL of extract was injected into the HPLC system. Characteristic MRM (multiple reaction mode) fragmentation ions were used for the identification and quantitation of phenolic acids: sinapic, benzoic, chlorogenic, *p*-hydroxybenzoic, caffeic, syringic, *p*-coumaric, and ferulic. The commercially available standards were used to prepare the calibration curves (Sigma-Aldrich, St. Louis, MO, USA). Contents of the phenolic acids in the raw material were calculated against a base of calibration curves plotted as the dependence of the area surface under the peaks for the standard phenolic acids.

### 3.7. Antioxidant Enzyme Activity Analysis 

Catalase (CAT), peroxidase (POD), and superoxide dismutase (SOD) were measured spectrophotometrically using a microplate reader. One hundred milligrams of fresh plant tissue was homogenised at 4 °C with 1.5 mL of 0.05 M potassium phosphate buffer (pH 7.8) containing 0.01 M EDTA (Avantor Performance Materials Poland SA, Gliwice, Poland). The homogenate was centrifuged at 15,000 rpm for 15 min. CAT activity was measured in 200 µL of supernatant mixed with 50 µL of 0.03 M H_2_O_2_ (Sigma-Aldrich, St. Louis, MO, USA) and estimated at *λ* = 240 nm by calculating the rate of H_2_O_2_ decomposition, according to Aebi’s [74] method. POD activity was measured in 200 µL of supernatant, as the number of oxidation products of 1% p-phenylenediamine (Sigma-Aldrich, St. Louis, MO, USA) (5 µL) in the presence of H_2_O_2_ (5 µL) at *λ* = 485 nm [75]. For determination of SOD activity, 5 µL of supernatant was combined with 200 µL of 0.05 M potassium phosphate buffer containing 1 M cytochrome c (Sigma-Aldrich, St. Louis, MO, USA) and 1 M xanthine (Sigma-Aldrich, St. Louis, MO, USA) (aerated for 12 h in the dark before use) and 5 µL of xanthine oxidase (Sigma-Aldrich, St. Louis, MO, USA). SOD activity was measured by McCord and Fiodovich’s [76] cytochrome method at *λ* = 550 nm and defined as the amount of enzyme necessary to inhibit cytochrome c in a coupled system with xanthine and xanthine oxidase. The enzymatic activity was converted into the amount of protein present in the plant tissue, according to Bradford’s [77] dye-binding method, using bovine serum albumin as a protein standard.

### 3.8. Amaryllidaceae Alkaloids Analyses

The alkaloids were extracted from lyophilised plants (150 mg of powder) by maceration for 24 h with 60% methanol (Sigma-Aldrich, St. Quentin Fallavier, France) (10 mL) and sonication at room temperature for 90 min in an ultrasonic bath (Transsonic 460/H Elma, Singen/Hohentwiel, Germany). After centrifugation at 4000 rpm for 20 min (Rotofix 32A, Hettich, Germany), the mixture was purified using the solid-phase extraction (SPE) cartridges and analysed by the GCMS-QP2010 Shimadzu equipment (Shimadzu, Kyoto, Japan), as previously described by Spina et al. [78]. The identification of the alkaloids was performed by comparing the measured data with those of the authentic compounds (galanthamine and lycorine), or with previous data [79]. The alkaloids were quantified using LC-MS equipment constituted by U3000-Dionex and micrOTOF-Q™ (Bruker Daltonics, Bruker, Bremen, Germany). An internal standard calibration method, along with a nine-point calibration curve (R^2^ = 0.99) using authentic galanthamine and lycorine (Sigma-Aldrich, St. Quentin Fallavier, France), was used for quantitative analysis of alkaloids. The analysis for the quantification of alkaloids was repeated three times.

### 3.9. Data Analysis

Statistical analysis of the data was performed with analysis of variance. Differences between the means were determined using Duncan’s multiple range test at *p* < 0.05. The values shown are the means ± standard deviation (SD).

## 4. Conclusions

In conclusion, we have demonstrated, for the first time, the influence of different light conditions on *L. aestivum* plants in vitro through the analysis of biomass, primary and secondary metabolite productions, and antioxidant enzyme activities. White-red and red LED lights promoted biomass growth, while blue LED light was demonstrated to be optimal to produce Amarylliadceae alkaloids. In terms of phenolic acids, the effects of differently coloured LED lights were specific to certain acids, with white-red and blue LED lights playing key roles. The results also suggest that LED lights cause a specific stress, which is manifested in CAT and SOD activities as well as secondary metabolite biosynthesis. Based on this study, the next steps in optimising the biomass growth alongside the production of phenolic acids and Amaryllidaceae alkaloids in *L. aestivum* in vitro cultures could be their cultivation in bioreactors under different proportions of a mixed white-blue-red LED light. 

It is worth emphasising that this initial identification of phenolic acids in *L. aestivum* cultures can create new opportunities for further research into the optimisation of the biosynthesis of these medically important metabolites using elicitation techniques.

## Figures and Tables

**Table 1:** Effect of different light conditions on photosynthetic pigment (chlorophyll *a*, *b*, carotenoids) concentrations in extracts of *L. aestivum* in vitro cultures. The results are means of 3 replicates (*n* = 3) ± SD. Different letters indicate a significance difference at *p* < 0.05 according to ANOVA and Duncan’s test. F: fluorescence, L: LED light, FW: fresh weight.

Photosynthetic Pigment Concentrations (µg/g FW)	Light			
	White (F) (Control)	Blue (L)	Red (L)	White (L)-Red (L)
Chlorophyll *a*	14.13 ± 1.53 ^ab^	16.01 ± 0.98 ^a^	13.06 ± 0.72 ^b^	8.51 ± 1.13 ^c^
Chlorophyll *b*	12.85 ± 0.39 ^a^	11.64 ± 2.14 ^a^	11.11 ± 1.95 ^a^	11.12 ± 1.10 ^a^
Carotenoids	2.24 ± 0.52 ^a^	1.82 ± 0.13 ^ab^	1.27 ± 0.04 ^b^	0.37 ± 0.16 ^c^

**Table 2:** Effect of different light conditions on total sugars, phenolic compounds and flavonoids contents in *L. aestivum* in vitro plants. The results are means of 3 replicates (*n* = 3) ± SD. Different letters indicate a significance difference at *p* < 0.05 according to ANOVA and Duncan’s test. F: fluorescence, L: LED light, DW: dry weight.

Total Content of Metabolites	Light			
	White (F) (Control)	Blue (L)	Red (L)	White (L)-Red (L)
Sugars [mg/g DW]	73.93 ± 2.54 ^a^	72.57 ± 1.23 ^a^	70.63 ± 1.14 ^a^	70.22 ± 4.75 ^a^
Phenolic compounds [mg/g DW]	51.52 ± 0.27 ^a^	49.92 ± 1.91 ^a^	48.58 ± 2.32 ^a^	51.07 ± 0.56 ^a^
Flavonoids [µg/mg DW]	0.40 ± 0.03 ^a^	0.32 ± 0.01 ^ab^	0.29 ± 0.01 ^b^	0.36 ± 0.05 ^ab^

**Table 3:** Phenolic acid concentrations in extracts of *L. aestivum* in vitro cultures. The results are means of 3 replicates (*n* = 3) ± SD. Different letters indicate a significance difference at *p* < 0.05 according to ANOVA and Duncan’s test. F: fluorescence, L: LED light, DW: dry weight.

Phenolic Acid Concentrations [ng/mg DW]	Light			
	White (F) (Control)	Blue (L)	Red (L)	White (L)-Red (L)
chlorogenic	0.06 ± 0.003 ^a^	0.06 ± 0.006 ^a^	0.06 ± 0.004 ^a^	0.05 ± 0.001 ^a^
*p*-hydroxybenzoic	0.67 ± 0.044 ^c^	1.07 ± 0.039 ^a^	0.82 ± 0.034 ^bc^	0.95 ± 0.114 ^ab^
caffeic	1.88 ± 0.175 ^b^	1.80 ± 0.036 ^b^	2.04 ± 0.191 ^b^	3.16 ± 0.153 ^a^
syringic	0.36 ± 0.027 ^b^	0.37 ± 0.018 ^b^	0.28 ± 0.007 ^c^	0.48 ± 0.007 ^a^
*p*-coumaric	1.30 ± 0.004 ^a^	1.06 ± 0.015 ^b^	1.18 ± 0.087 ^ab^	1.18 ± 0.016 ^ab^
ferulic	3.07 ± 0.206 ^b^	2.98 ± 0.085 ^b^	2.70 ± 0.065 ^b^	3.54 ± 0.145 ^a^
sinapic	1.98 ± 0.082 ^c^	2.62 ± 0.064 ^a^	1.70 ± 0.055 ^d^	2.20 ± 0.057 ^b^
benzoic	1.66 ± 0.161 ^c^	2.83 ± 0.215 ^a^	2.24 ± 0.055 ^b^	2.08 ± 0.079 ^bc^

**Table 4:** Effect of different light conditions on (**a**) catalase (CAT), (**b**) peroxidase (POD) and (**c**) superoxide dismutase (SOD) activities in *L. aestivum* in vitro plants. The results are means of 3 replicates (*n* = 3) ± SD. Different letters indicate a significance difference at *p* < 0.05 according to ANOVA and Duncan’s test. F: fluorescence, L: LED light.

Antioxidant Enzyme Activities (U/mg protein)	Light			
	White (F) (Control)	Blue (L)	Red (L)	White (L)-Red (L)
CAT	0.38 ± 0.15 ^b^	0.45 ± 0.07 ^ab^	0.69 ± 0.13 ^a^	0.51 ± 0.13 ^ab^
POD	4.33 ± 0.37 ^a^	4.03 ± 0.42^a^	4.02 ± 0.32 ^a^	4.47 ± 0.10 ^a^
SOD	0.14 ± 0.01 ^b^	0.12 ± 0.01 ^b^	0.20 ± 0.05 ^a^	0.14 ± 0.01 ^b^

**Table 5:** Amaryllidaceae alkaloids identified by GC-MS (% of TIC) in *L. aestivum* in vitro plants grown in different light conditions. TIC: Total Ion Chromatogram, F: fluorescence, L: LED light, nd: not detected, (-): no alkaloid.

Alkaloid	Formula	Retention Time [min]	Base Peak	Light			
				White (F) (Control)	Blue (L)	Red (L)	White (L)-Red (L)
Galanthamine	C_17_H_21_NO_3_	9.30	287	nd	nd	nd	nd
Demethylmaritidine	C_16_H_19_NO_3_	10.74	273	15.3%	21.4%	-	20.6%
Lycorine	C_16_H_17_NO_4_	14.03	226	15.6%	15.65%	nd	nd

## Data Availability

The data that support the findings of this study are available from the corresponding author upon reasonable request.
